# Revisiting the Role of Sensors for Shaping Plant Research: Applications and Future Perspectives

**DOI:** 10.3390/s24113261

**Published:** 2024-05-21

**Authors:** Anshika Tyagi, Zahoor Ahmad Mir, Sajad Ali

**Affiliations:** 1 Department of Biotechnology, Yeungnam University, Gyeongsan 38541, Republic of Korea; 2 Department of Plant Science and Agriculture, University of Manitoba, Winnipeg, MB R2M0TB, Canada; zahoorbio@gmail.com

**Keywords:** sensors, nanosensors, plant stress biology, signaling molecules, environmental stressors, smart agriculture, precise agriculture

## Abstract

Plant health monitoring is essential for understanding the impact of environmental stressors (biotic and abiotic) on crop production, and for tailoring plant developmental and adaptive responses accordingly. Plants are constantly exposed to different stressors like pathogens and soil pollutants (heavy metals and pesticides) which pose a serious threat to their survival and to human health. Plants have the ability to respond to environmental stressors by undergoing rapid transcriptional, translational, and metabolic reprogramming at different cellular compartments in order to balance growth and adaptive responses. However, plants’ exceptional responsiveness to environmental cues is highly complex, which is driven by diverse signaling molecules such as calcium Ca^2+^, reactive oxygen species (ROS), hormones, small peptides and metabolites. Additionally, other factors like pH also influence these responses. The regulation and occurrence of these plant signaling molecules are often undetectable, necessitating nondestructive, live research approaches to understand their molecular complexity and functional traits during growth and stress conditions. With the advent of sensors, in vivo and in vitro understanding of some of these processes associated with plant physiology, signaling, metabolism, and development has provided a novel platform not only for decoding the biochemical complexity of signaling pathways but also for targeted engineering to improve diverse plant traits. The application of sensors in detecting pathogens and soil pollutants like heavy metal and pesticides plays a key role in protecting plant and human health. In this review, we provide an update on sensors used in plant biology for the detection of diverse signaling molecules and their functional attributes. We also discuss different types of sensors (biosensors and nanosensors) used in agriculture for detecting pesticides, pathogens and pollutants.

## 1. Introduction

Modern agriculture faces several challenges like environmental stressors, climatic instability, labor shortages, and poor soil as a result of inadequate land management, which all affect agriculture productivity [1,2]. However, with the advent of sensors, many of these problems that plants face can be managed by their early detection. Modern agriculture has transformed into smart farming with the advancement in sensor-based technology [3,4]. The application of sensors (nanosensors and biosensors) that are capable of sensing changes in plant health and their morphological and physiological traits has shown to be a useful technique to boost agricultural yields [5]. Sensors used in agriculture may gather information about crops, fields, the environment, soil, and other vital characteristics, allowing farmers and other agricultural professionals to make the best decisions on how to manage their crops and fields [5,6]. In smart farming, sensor-based phenotyping has revolutionized plant research by monitoring different growth and stress traits of crops grown under different conditions [7]. Sensors play a multifaceted role to monitor different plant signaling molecules and environmental stressors in real time, which has paved the way for improving plant growth and crop yield. They also play a key role in monitoring soil variables like pH, pesticides, heavy metals, moisture, and minerals, in real time. In plant biology research, different types of sensors such as biosensors and nanosensors are used which are made up of different materials.

**Figure 1:**
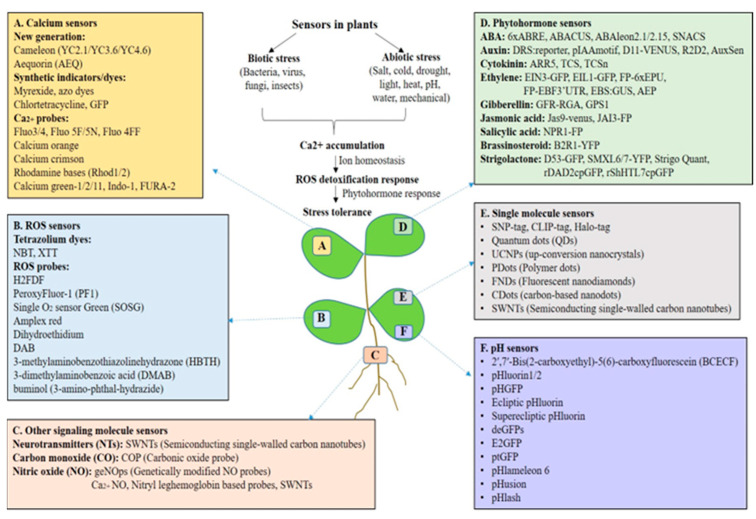
Sensors used in plant biology for the detection of signaling molecules such as calcium (Ca^2+^), ROS, hormones, and nitric oxide (NO) which regulate diverse growth and stress responses. This Figure also shows different sensors used for monitoring pH in plants.

Plants’ sessile nature necessitates that they recognize environmental obstacles that may threaten their survival. Individual cells that detect possible hazards to neighboring cells must signal and communicate quickly in order to survive. In response to environmental stressors, plants produce diverse signaling molecules such as Ca^2+^, ROS, hormones, etc., which regulate diverse growth and adaptable responses [8]. For instance, Ca^2+^ is a well-known secondary messenger and signaling element present in both unicellular and multicellular organisms. Biotic and abiotic stresses alter cellular Ca^2+^ homeostasis by causing transitory fluctuations in Ca^2+^ concentrations in the cytosol and subcellular compartments, which are further sensed by different Ca^2+^ sensors regulating different growth and stress-related responses [9]. Ca^2+^ binding proteins such as calmodulin (CaM), CaM-like proteins (CMLs), and calcineurin B-like proteins (CBLs), detect changes in Ca^2+^ levels. This causes the proteins to alter shape, allowing them to interact with numerous targets and regulate downstream processes including transcription, enzymatic activity, and ion fluxes [10,11]. Plants feature particular Ca^2+^ active transporters like Ca^2+^ -ATPases and Ca^2+^/cation exchangers (CAX), which are present in cell membrane and intracellular organelles and contribute to the replenishment of resting [Ca^2+^] cyt. This cellular Ca^2+^ signature or cellular dynamics can be observed utilizing Ca^2+^ imaging methods. Earlier synthetic dyes that were Ca^2+^-sensitive (e.g., Ca^2+^ green dextran, Fura-2, Fura-2 dextran, and Indo-1) were used for the detection of cytosolic Ca^2+^ dynamics by fusing with Ca^2+^-selective chelators like EGTA and BAPTA, respectively. However, because of their limitations, the introduction of fluorescent-based genetically encoded Ca^2+^ indicators (GECIs) such as Aequorin, cameleon, and YC-Nano offered additional opportunities for the quantitative in vivo imaging of Ca^2+^ dynamics [12]. Additionally, imaging can be used to detect Ca^2+^ dynamics in different subcellular compartments, such as the tonoplast, the nucleus, the Golgi apparatus, mitochondria, plastids/chloroplasts, apoplastic space, and the thylakoid lumen and membrane [13,14]. Furthermore, the bioluminescence resonance energy transfer (BRET)-based GFP-aequorin reporter or sensor (i.e., G5A) solved the primary constraints of aequorin, which permitted imaging of long distance Ca^2+^ waves with a lower quantity of emitted light [15]. Similarly, Yellow Cameleon (YC) was developed using GECIs followed by a series of variants with higher affinity for Ca^2+^ as shown in Figure 1. Modern research has led to the development of highly sensitive single-fluorophore (single-FP) biosensors (GCaMP biosensors) connected with calmodulin, which have several advantages over FRET Cameleons. These include a simpler experimental design and possibly higher temporal resolution of imaging.

ROS also operate as secondary messengers in plant signal transduction altering diverse plant growth and stress-related traits. They have important roles in regulating a wide range of subcellular, cellular, and systemic signals. Additionally, ROS are vital in plant defense and acclimation responses to various biotic and abiotic environments, as well as being a critical component of several hormonal, physiological, and developmental pathways [16,17,18]. The real time monitoring of ROS in plants reveals the molecular complexity of plant signal transduction pathways related to different traits. Many sensors and reporters are used to monitor live ROS imaging in plants such as 2′,7′-dichlorofluorescin diacetate (H_2_DCFDA), H_2_DCFDA conjugated to BSA (OxyBURST), dihydroethidium (DHE) and its mitochondrion-targeted form mitoSOX, dihydro-2′,4,5,6,7,7′-hexafluorofluorescein (H_2_HFF), N-acetyl-3,7-dihydroxyphenoxazine (Amplex red), singlet oxygen sensor green (SOSG), and boronate-based probes such as peroxy orange-1 (PO1) [17]. These detection systems vary in detecting different ROS forms in plants.

Plant hormones play a key role in plant defense–growth tradeoffs and are key for plant survival [18]. Phytohormones are natural compounds, which control various physiological processes during stress as well as growth and development. A wide range of growth hormones [auxin (AUX), cytokinin (CK), and gibberellin (GB)] and stress hormones [abscisic acid (ABA), jasmonic acid (JA), salicylic acid (SA), ethylene (ET), brassinosteroids (BRs), strigolactones (SLs), and small peptides] have been characterized in plants [19,20]. The hormone-signaling network is very complex and interconnected. Earlier, the traditional biochemical methods such as immunohistochemistry, LC-MS or GC-MS were used to check the distribution, quantity, and identification of these hormones in plant cells and tissues [21], at high accuracy and sensitivity [22]. The advances in synthetic biology based on sensors have paved the way to broaden our knowledge of the distribution of plant hormones in different tissues, organs, and cell types. Synthetic biology offers continuous monitoring, live cell imaging, and identification of local distribution of phytohormonal concentration in plants under normal and various stress conditions. New techniques for detecting phytohormones with minimum invasion and cellular or even subcellular resolution have been made possible over the past 20 years by developments in fluorescence microscopy technology and biosensor engineering [23]. For example, to visualize AUXtransport dynamics and subcellular AUX distribution, fluorescent conjugates of AUX, such as 7-nitro-2,1,3-benzoxadiazole (NBD)-naphthalene-1-acetic acid (NAA) and NBD-indole-3-acetic acid (IAA) have been used in the model plants. Alexa Fluor 647-castasterone (AFCS) was utilized to observe BR receptor endocytosis in live Arabidopsis cells [24]. Similarly, bioactive fluorescein-labeled gibberellic acids (GA-fls) and the strigolactone (SL)-agonist probe Yoshimulactone Green (YLG) becomes activated and so fluorescent upon GA and SL application [25]. Peptide hormones were also detected using fluorescent dyes such as tetramethylrhodamine (TAMRA) to monitor their uptake and localization [26]. Another form of FRET sensor, notably ABACUS and ABAleon, a SnRK2 activity sensor (SNACS), was created and utilized to monitor ABA buildup [27]. A two-component output-sensor (TCS) was designed as a synthetic reporter to visualize the distribution of CK in Arabidopsis embryos, and its enhanced derivative, TCS-new (TCSn), was also developed. EIN3-GFP and EIL1-GFP were developed as sensors for ET. Vong et al. [28] created an enzyme-based chemical biosensor called the artificial-metalloenzyme ethylene probe (AEP) to detect ET. In addition, GPS1 which is a FRET-based GA sensor was designed to measure spatiotemporal GA distribution with high resolution [29]. Furthermore, fast changes in the quantity and distribution of plant hormones targeting specific compartments in living cells can be detected by genetically encoded biosensors (direct or indirect) [30].

The role of Ca^2+^ and ROS signaling responding to various external stimuli in plants is well studied but the physiological significance of pH changes is largely unknown. However, reports on pH-sensing studies based on leaf and root tissue led to the discovery of systems that sense and signal extracellular pH (pHe). In plants, pH regulates the chemistry and rheology of the cell wall to change its flexibility and govern the spatiotemporal growth of cells. pHe homeostasis is cooperatively maintained by cell wall components, enzymes that remodel the cell wall, and H^+^-ATPases located in the plasma membrane (PM). The pHe of plants is inherently acidic, but it fluctuates dynamically in response to environmental stimuli and physiological factors [31]. In general, use of fertilizer, the climate, and weather can vary the plant pH externally (soil), leading to a change in apoplastic pH [32], but inside, the apoplastic pH is also altered by defense and growth activities [33,34]. Recent findings demonstrate that transmembrane kinase1 (TMK1) phosphorylates and activates plasma membrane H^+^-ATPases, which causes apoplastic acidification, therefore modulating the AUX signal that drives cell elongation in the hypocotyl and root elongation zones [33,34]. In plants, a change in apoplast pH or extracellular alkalization is the first and most immediately observable reaction during pattern-triggered immunity (PTI), drought and salinity [35]. The secondary regulation of proton pumps or the passage of ions cause an elevation in systemic pH in plants [31]. In addition, Ca^2+^ transients lead to pH changes in the cytosol for distinct stimuli known as the pH–Ca^2+^ link. To fully comprehend the relationship between cytosolic Ca^2+^ transients and H^+^ homeostasis, various fluorescent biosensors [notably, NES-YC3.6 and pH-green fluorescent protein (GFP)] have been used to analyze pH and Ca^2+^ dynamics in living plant cells [36,37].

In plants, numerous GFP variations exhibit pH sensitivity due to chromophore protonation and deprotonation [38]. For example, various cytosolic and other organelle GFP-based biosensors such as pHluorin and Pt-GFP, as well as H148D pH sensors give a comprehensive set of tools for imaging fluctuations in these ions. These fluorescent probes have the limitation that they work only in cytoplasm. As a result, a genetically encoded pH indicator overcomes many of the disadvantages of traditional chemical probes. Recently, the Fluorescence Indicator reporting pH in Lysosomes (FIRE-pHLy) has been used to target lysosomal pH [39]. The use of pH-sensitive GFPs has resulted in the rather surprising finding that, rather than a tightly buffered, constant cytosolic pH, cells can display extremely dynamic pH variations in response to a wide variety of internal and external stimuli. Such pH fluctuations should have extensive impacts on cellular biochemistry, implying that pH, together with redox, might operate as a worldwide coordinator of cell activities, moving the balance of the cell between signaling/response states [38]. Biosensors are currently advancing the frontiers of our understanding of the in vivo cellular dynamics that underpin important regulatory networks at dimensions from the subcellular to the entire plant [38]. To summarize, we have shown different sensors used in plant research for detecting different signaling molecules like Ca^2+^, ROS, hormones, nitric oxide (NO), neurotransmitters (NTs) and pH in Figure 1.

## 2. Application of Nanosensors in Agriculture

**Figure 2:**
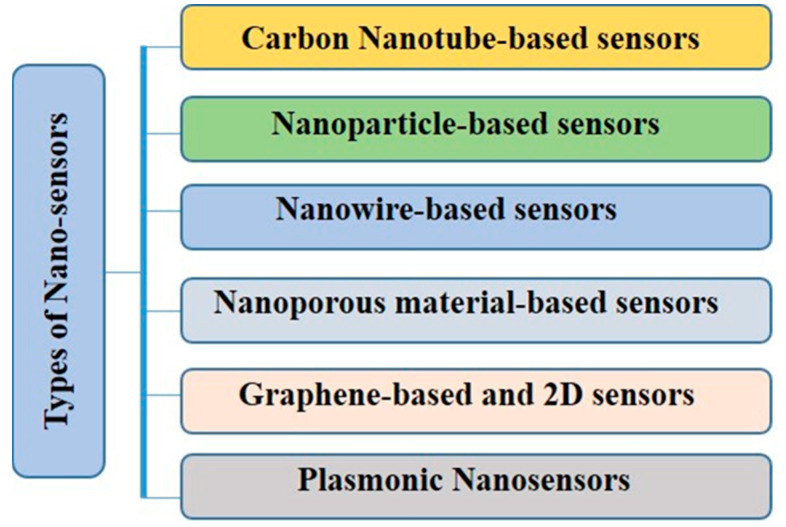
Types of nanosensors made of different materials used for the detection of diverse molecules.

Owing to their unmatched capability to sense and response to a broad spectrum of environmental stimuli and exceptional sensitivity and specificity, nanosensors have become extremely useful tools for the real-time monitoring and management of plants. Nanobiosensors are non-intrusive, sensitive devices that are developed using combined nanobiotechnological approaches to monitor a large number of environmental samples [40]. Nanobiosensors collect the information for analysis and produce signals in real-time response [41]. Different types and classes of nanomaterials including nanotubes (multi-walled and single-walled), one-dimensional nanowires, quantum dots, crystalline particles also known as nanocrystals, nanoceramics and nanocomposites and hybrid materials can be exploited to manufacture nanobiosensors. The different types of nanosensors that are used in agriculture are shown in Figure 2.

**Figure 3:**
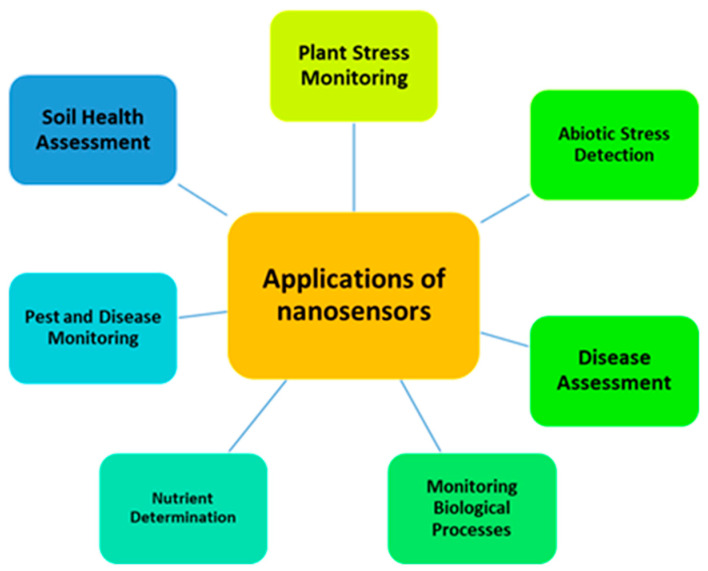
The applications of nanosensors for the detection of biological processes, nutrients, and biotic and abiotic stressors, as well as soil health monitoring and disease assessment.

Nanobiosensors have an immense range of applications from household to industry which includes detection of a wide variety of fertilizers, pesticides, fungicides, pathogens, heavy metal content, and quality of soil such as pH, temperature, moisture content, soil water content and overall growth hormone level [42,43,44]. In addition, farmers utilize these portable smart sensors to monitor and manage the soil conditions locally in the agricultural industry. They record the concentrations of minerals, thus insufficiencies of particular minerals in the soil, and detection of pests, pathogens, and diseases [45]. We have shown the application of sensors in agriculture in Figure 3.

## 3. Application of Nanosensors for Pesticide Detection

Various organic and inorganic substances, including dyes, pesticides, and agricultural wastes containing hazardous materials, are introduced into soil through agricultural activities, industrial wastewater, and municipal wastewater [46]. It is essential to monitor xenobiotics in soil, particularly herbicides and soil-applied pesticides, to prevent their uptake by plants, given their detrimental effects on the environment and living organisms [47]. Due to limitations and challenges associated with conventional analytical methods for pesticides, there is a growing interest in developing new measurement techniques such as biosensors and nanobiosensors [48]. Nanosensors are small devices designed to detect specific molecules, biological components, or environmental conditions at the nanoscale [49]. They offer high specificity, portability, and superior detection capabilities compared to larger sensors [50]. Nanosensor operation typically involves three main components: sample preparation, recognition of target molecules or organisms, and signal transduction [51]. Recognition molecules such as antibodies or enzymes bind to target analytes in the sample, and signal transduction methods convert these interactions into measurable signals [45]. This allows for precise and efficient detection of a wide range of substances and environmental factors.

Significant progress has been made in the last several decades in the development of nanomaterial-based sensors for the detection of pesticide residues in soil [51]. Detecting pesticide residues is crucial for ensuring food safety, particularly in animals, as low concentration pesticides like organophosphates can accumulate [52]. Exposure to higher concentrations poses severe health risks by inhibiting enzymes such as acetylcholinesterase [53]. Thus, the development of advanced detection methods is paramount for safeguarding human health and maintaining food safety standards. Nanosensors offer innovative solutions for detecting various pesticide residues with high sensitivity and selectivity [54]. Nanoparticles serve two primary functions in these systems: signal conversion and signal enhancement. Signal conversion involves nanoparticles altering color or emitting light in response to pesticide presence, while signal enhancement utilizes nanoparticles to boost detection sensitivity through various means such as enhancing fluorescence or Raman signals [51]. Various kinds of nanosensors differing in their sensing ability to detect the herbicide, insecticide and pesticide residues within the soil samples have been introduced recently including paper-based screen-printed electrodes (SPEs) with nanostructure modifications on the transducer surface which improves both the portability and sensitivity of the electrochemical detection platform [55]. Moreover, a fluorescent nanosensor has been introduced utilizing Ytterbium Oxide nanoparticles, which have been modified using 3-aminopropyl-triethoxysilane and coated with Yb_2_O_3_, enabling the detection of imazapyr with a limit of detection of 0.2 ppm. This sensor exhibits proficient capabilities in sensing herbicides effectively [56]. Another study introduces an affinity sensor based on surface plasmon resonance (SPR) which utilizes atrazine-imprinted nanoparticles attached to a gold surface for the selective detection of atrazine, with a detection limit of 0.7134 ng/mL, while yet another SPR-based fiber-optic sensor incorporates tantalum (V) oxide (Ta_2_O_5_) nanoparticles for detecting fenitrothion with a detection limit of 38 nM [57]. Nanomaterials show considerable promise for developing non-enzymatic electrochemical sensors. These include various categories such as nanoparticles (e.g., CuO, CuO–TiO_2_, ZrO_2_, and NiO); nanocomposite (e.g., molybdenum); and peptide and carbon nanotubes, which are extensively utilized for electrochemically detecting residual pesticide particles [58]. The thorough study of residual pesticide particles using such nanomaterials is attributed to their extremely small size, large surface area, and unique electrical and chemical properties.

Recently, there has been an increasing focus on utilizing nanomaterials to improve the performance of electrode surfaces in the detection of heavy metals [59]. Electrodes modified with nanomaterials have demonstrated significant advancements in electroanalytical techniques for detecting a diverse array of heavy metals. These nanomaterials encompass metal nanoparticles, metal oxides, graphene-based materials, carbon nanotubes, and metal–organic frameworks (MOFs) [60]. Common methods for fabricating nanomaterial-modified disposable electrodes include drop casting, dip coating, spin coating, electrochemical deposition, direct growth, and screen printing [61]. One fluorescence sensor utilizes a combination of copper (II) oxide and multiwall carbon nanotubes (MWCNTs) to monitor glyphosate, achieving a limit of detection of 0.67 ppb [62]. Meanwhile, an electrochemical luminescence sensor detects glyphosate at 0.5 nM by employing composites of luminol–gold nanoparticles–L-cysteine–Cu (II) [63]. Moreover, an electrochemical sensor employing CuO-TiO_2_ hybrid nanocomposites detects methyl parathion at 1.21 ppb [64], while an electrochemical aptasensor detects malathion at 0.001 ng/mL utilizing CuO nanoparticle-decorated 3D graphene nanocomposites [65]. Optical nanosensors utilizing silver nanodendrites detect dimethoate at 0.002 ppm [66], and upconverting nanoparticles detect metribuzin at 6.8 × 10^−8^ M through ratiometric and colorimetric responses [67]. With a limit of detection of 0.01 nM in soil samples, nanosensors showcase the potential for accurate, rapid, and dependable detection of pesticide residues, thereby aiding environmental monitoring and ensuring environmental safety. Different types of nanosensors used for the detection of pesticides are shown in Table 1. Future efforts should prioritize developing simplified detection methods, including miniaturized and intelligent approaches such as colorimetry and test papers. Furthermore, there is a need to expand detection capabilities to encompass a wider range of pesticide types and enable simultaneous detection of multiple pesticides using high-throughput chip-based technologies combined with nanomaterials.

## 4. Application of Nanosensors for the Detection of Heavy Metals

Heavy metal pollution poses serious environmental threats, coming from industrial activities, urban runoff, and human practices. Heavy metals have the potential to bioaccumulate and biomagnify in living organisms through the food chain, posing significant health risks [70]. Studies have documented various acute and chronic toxic effects of heavy metal ions on human organs [71,72]. Exposure to heavy metal contamination can lead to oxidative stress, ecological toxicity, plant toxicity, morphological and biochemical effects, and cellular toxicity in the living organisms [66]. Moreover, increased levels of heavy metals in humans have been associated with numerous health hazards, including lower IQ in children, developmental obstacles, cancers, hypertension, weakened immune systems, cellular toxicity, oxidative damage, heart diseases, coronary artery disease, cerebrovascular disorders, and miscarriages and stillbirths, among others [72,73]. Recent incidents such as the lead tap water crisis in Flint, Michigan, highlight the critical need to be prepared for potential widespread heavy metal contaminations and the associated health risks, social consequences, and post-traumatic stress disorders [74,75].

Due to its sensitivity and convenience, electrochemical detection, especially through portable and disposable sensors, has emerged as a powerful method for monitoring heavy metals [76]. Nanomaterials, such as various oxides of metal nanoparticles, graphene-based materials, carbon nanotubes, and metal–organic frameworks (MOFs), play a crucial role in enhancing electrode surfaces for monitoring heavy metals [41]. Various nanostructure architectures have shown promising results in detecting heavy metals with high sensitivity. Metal–organic frameworks (MOFs) offer precise pore sizes and functional groups for selective sensing [77]. Chemically modified silicon membranes, electrodeposited bismuth films, and ion-imprinted polymer films have all been employed for the efficient and accurate detection of heavy metals within environmental samples [78]. These advancements underscore the potential of nanomaterial-based electrochemical sensors for effective monitoring and management of heavy metal pollution.

Significant achievements have been achieved in the advancement of analytical procedures for detecting and analyzing heavy metal ions (HMIs) in environmental samples. For heavy metal analysis, well-established methods such as graphite furnace atomic absorption spectroscopy (GF–AAS), flame atomic absorption spectroscopy (FAAS), inductively coupled plasma–mass spectroscopy (ICP–MS), atomic emission spectroscopy (AES), inductively coupled plasma–optical emission spectroscopy (ICP–OES), X-ray diffractometry, and X-ray fluorescence have been identified [79,80,81]. However, these techniques often come with drawbacks such as large sample sizes, expensive equipment, and the need for specialized training, limiting their practicality for on-site or field studies. Electrochemical and colorimetric nanosensors have emerged as promising alternatives due to their high sensitivity, specificity, affordability, mobility, and rapid detection capabilities [82]. Various electrochemical nanosensors have gained attention for their ability to identify heavy metal ions with high efficiency and specificity, making them appropriate for on-demand, in situ, and field applications [83,84]. Similarly, colorimetric nanosensors enable quick screening and visual detection for point-of-use applications. Recent reviews have highlighted the advances in nanomaterial-based optical sensors for heavy metal detection, with a focus on affordable and compact electrochemical nanosensors and smartphone-operated screen-printed electrodes (SPEs), among other technologies [85]. Developing reliable methods for heavy metal detection for environmental samples is crucial for ensuring public health safety and global homeland security.

Significant studies have been undertaken to generate sensors that are efficient in detecting heavy metal ions at trace and ultra-trace concentrations [86,87]. However, challenges such as sensitivity, selectivity, specificity, and interference persist in many available sensors [88,89]. Therefore, considerable efforts must be directed towards enhancing electrochemical and colorimetric nanosensors to achieve better efficiency, accuracy, and specificity, thus enabling reliable and expanded heavy metal ion detection capabilities [90,91]. Specifically, advancements should focus on designing and developing easily movable electrochemical sensors based on graphene, carbon nanotubes, nanostructures, carbon dots, nanomaterials, and metal–organic frameworks to facilitate accurate and specific heavy metal ion detection [92]. The advancement of portable colorimetric sensors for rapid screening and visual detection of heavy metal ions will continue to be an active area of research in the coming years [93]. Enhanced technology coupled with smartphone accessibility will open avenues for widespread adoption and enhancement of smartphone-based sensors, enabling rapid, in situ, and on-site detection of heavy metal ions [94]. We have summarized the use of various sensors for the detection of heavy metals in Table 2. Furthermore, the emergence of low-cost and disposable paper-based sensors will facilitate on-site and field detection of heavy metal ions. There will be a growing interest in microfluidic and microchip sensors to enable rapid arrays and simultaneous detection of heavy metal ions. The utilization of functionalized gold nanoparticles for fiberoptic surface plasmon resonance sensing of heavy metal ions is projected to attract great attention.

## 5. Role of Nanosensors for the Detection of Phytopathogens and Pests

**Figure 4:**
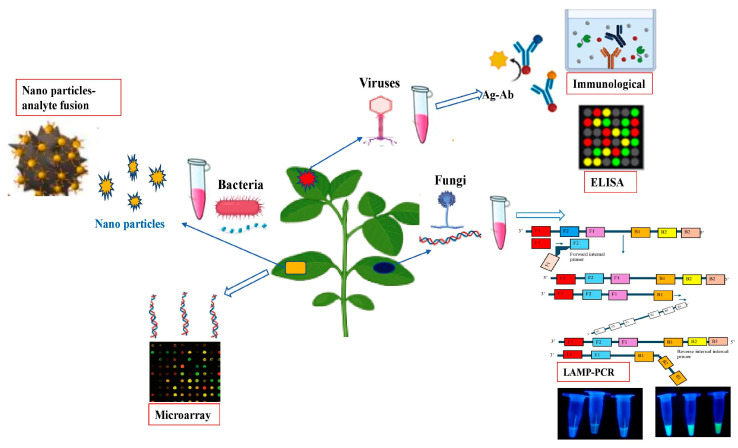
Schematic illustration of different nanosensor-based pathogen detection systems in plants.

The world economy is still at risk from emerging plant diseases that continue to be a serious threat to food security and ecological stability. Plant pathogens pose a significant threat to global agriculture, with potential yield losses of up to 30% [18,102]. Fungal diseases cause enormous agricultural losses, hence effective management measures must be taken to prevent further infestation. The introduction of new technologies in disease detection and diagnosis has increased agricultural output by enabling real-time identification of plant diseases at an early stage of infection. Plant health monitoring increasingly requires early identification of plant pathogens in order to control diseases at various stages of development, reduce the risk of spreading, and prevent the entry of novel pathogens. Traditional procedures are deemed to lack the precision, accuracy, and sensitivity required to identify plant diseases. Conventional diagnostic methods use time-consuming, culture-dependent procedures that are especially difficult when it comes to dealing with biotrophic fungal pathogens. Traditional methods of plant pathogen identification mainly rely on descriptive methods that interpret visible symptoms in addition to isolation, culture, and laboratory-based methods that include physiological, biochemical, and pathogenicity tests [103]. The precision and reliability of these methods largely hinge on the expertise and proficiency of the person undertaking them. A new era in agricultural diagnostic technology was ushered in with the rapid advancement of molecular diagnostic techniques in recent decades. Plant disease identification has been made easier by the advent of novel molecular techniques which include the use of many types of the polymerase chain reaction (PCR), including nested PCR, multiplex PCR, reverse transcription (RT)-PCR, real-time PCR, and conventional PCR [104]. Critical immunological techniques include lateral flow assays (LFAs) [105], and enzyme-linked immunosorbent assays (ELISAs), plate-trapped antigen-ELISA, and double antibody sandwich-ELISA [106]. Despite these benefits, molecular detection techniques are not always able to identify pathogens at low titers in materials like seeds and insect vectors, or in the early stages of infection. Moreover, cross-contamination with PCR reagents that entirely prevent the target DNA amplification can result in false negative results, whilst cross-amplification of PCR-generated non-target DNA fragments can result in false positive results. The inability to use PCR for plant pathogen identification in the field is another restriction and thus necessitates the development of other viable detection tools [107]. In this regard, nanosensors have emerged as an important tool for the rapid detection of plant pathogens and pests in agriculture as shown in Figure 4. Different types of nanotechnology-based tools are used for plant pathogen detection which include nano-barcoding, nanobiosensors, metal nanoparticles, quantum dots, and nanodiagnostic kits. Nanomaterials such as nanoparticles, nanowires, and nanotubes have been extensively explored for the development of sensors with enhanced sensitivity and selectivity. Nanoscale sensors offer advantages such as high surface-to-volume ratios and increased signal amplification, enabling ultrasensitive detection of phytopathogens [108]. In recent times, gold nanoparticles and nanostructures have been used on a large scale for the detection of plant pathogens and disease diagnostics. For instance, *Xanthomonas axonopodis* pv. vesicatoria, the bacterium that causes bacterial spot disease in Solanaceae plants, has been identified using sensors that combine fluorescent silica nanoparticles with antibody molecules [109]. Similarly, phytoplasma was detected in grapes using gold nanoparticle-coated sensors [110]. In *Arabidopsis thaliana*, an electrochemical sensor coated with gold nanoparticles was used to detect the bacterial pathogen *Pseudomonas syringae* [111]. In addition to bacterial pathogens, nanosensors can also detect fungal pathogens mainly by detecting mycotoxins. For example, the biosensor 4mycosensor detects different types of fungal mycotoxins in different crops such as oat, corn, barley and wheat [112]. This nano-based diagnostic kit can assist farmers in preventing disease epidemics in the field. A previous study reported that *Aspergillus* spss can be accurately detected using copper oxide (CuO) nanoparticles [113]. In strawberry, carbon nanotubes were used to detect fungal pathogens such Rhizopus and Aspergillus species [114]. Another noteworthy detection method is based on surface-enhanced Raman spectroscopy (SERS), which is capable of recognizing chemical fingerprints. Using silver nanoparticles (AgNPs), this approach quickly detected Alternaria mycotoxins in pear fruit, with a limit of detection (LOD) of 1.30 μg/L [115]. For the detection of viral pathogens in plants, QD-based nanosensors have been used in different plant systems, for example, Grapevine virus A [116], Tomato ringspot virus [117], Cowpea mosaic virus [118], Cauliflower mosaic virus [119], Citrus tristeza virus [120], Bean pod mottle virus [117], and Arabis mosaic virus [117]. Sharma et al. [121] created a label-free immunosensor that detects Capsicum chlorosis virus (CaCV) in bell pepper leaves. This immunosensor was based on immobilizing viral antigens on the surfaces of gold nanoparticles (AuNPs) and multi-walled carbon nanotubes. Notably, the immunosensor had a sensitivity for CaCV detection that was 800–1000 times higher than that of DAC-ELISA. Gold nanorods (AuNRs) functionalized with antibodies specific to Cymbidium mosaic virus (CymMV) [122]. A new technique for detecting Citrus tristeza virus (CTV) using nanobiosensors was developed, which uses fluorescence emission from cadmium telluride quantum dots (CdTe-QDs) linked with CTV coat protein (CTV-CP) antibodies. Two sensitive detection approaches were discussed: Förster resonance energy transfer (FRET) biosensors and non-FRET-based biosensors for quick identification of CTV-infected plants [123].

In future, different nanoscale gadgets can be customized for smart agricultural systems to detect pathogens before growers become aware of them. Nano–smart devices will therefore act as a warning system and a safety measure for disease outbreaks. Nanoscale materials are useful tools for the detection of plant disease because of the remarkable biospecificity of synthetic molecular recognition at the nanoscale, which has lately seen enormous advancements.

Pest and disease-related crop losses pose a serious threat to global food security as well as to the earnings of farmers. Globally, pests cause up to 40% of the crop yield losses each year [124]. The proper prevention of plant pests can guarantee agricultural output mainly by preventing their development and their spreading in the fields. A great way to identify, anticipate, and control plant insect pests in different crops is by remote sensing utilizing nanosensors [125,126]. With a dimension of less than 100 nm, nanosensors are potent, real-time, cost-effective, and environmentally friendly sensing devices employed to detect insects. The use of the insect’s own pheromone is paving the way for developing nanosensor-based tools for pest detection [127]. For, instance, insect sexual pheromone, methyl 2,6,10-trimethyltridecanoate, was detected using a polyaniline and multi-walled carbon nanotube (Pani/MWCNT-COOH) nanocomposite, which is a nanostructured cantilever sensor [128]. In addition, a graphene oxide and β-cyclodextrinylated cantilever nanosensor was utilized to detect the presence of *Bactocera oleae* in olive plants. Wehrenfennig et al. [129] used a variety of metal-oxide gas sensors including tungsten and tin oxide nanoparticles to detect the sexual pheromone of the grapevine pest (*Lobesia botrana*). A previous study has demonstrated the effectiveness of an oxide film nanostructure and 3-aminopropyl triethoxysilane-functionalized silicon dioxide cantilever sensor in the detection of insects’ pheromones, particularly *Helicoverpa armigera* (Hubner) and *Scirphophaga incertulas* [130]. Brezolin et al. [131] developed an extremely sensitive nanostructured cantilever sensor for *Euschistus heros* pheromone detection, this being a major pest of soybeans. Similarly, a variety of metal-oxide gas sensors including tungsten and tin oxide nanoparticles were employed to detect the sexual pheromone of the grapevine pest (*Lobesia botrana*) [129]. These studies support the notion that nanotechnology-based sensors are viable tools for the early detection and monitoring of insects in sustainable agriculture which can reduce the usage of pesticides and boost yield production.

## 6. Challenges and Future Perspectives of Sensor-Based Smart Farming

The current agricultural sector has several noteworthy obstacles, such as the escalating need for food, scarcities of labor, environmental stressors and climate change. A viable solution for addressing the current issues facing the agriculture sector is that of smart farming and precision agriculture [132]. Smart agricultural farming uses modern technology, such as sensors, to increase crop yield while lowering adverse environmental consequences, increasing the sustainability of farming. From the farmers’ perspective and that of the environment, smart farming has several advantages. For example, smart farming methods use sophisticated sensors to monitor biotic and abiotic stressors, soil physicochemical properties and nutrient levels. Thus a farmer is helped to apply the proper quantity of inputs, such as fertilizer, water, and pesticides at the correct time and location by utilizing data and sensors to monitor crop conditions, weather, and soil. This can lower waste and pollution while increasing crop quantity and quality. In other words, smart farming can act as an early warning system, which can help farmers to monitors and identify possible problems and provide prompt remedies in order to avoid crop yield losses [133]. Although there are many advantages to smart farming, there are also many challenges to its broad implementation. For small-scale farmers, there may be several obstacles, including the initial cost of technology, data interpretation, data privacy, and incompatibilities with current agricultural practices. In order to overcome these obstacles, education and training initiatives must be put in place to provide farmers the know-how to properly utilize these technologies [134]. On the hand, the performance of sensors is frequently impacted by environmental conditions including humidity and temperature variations. In order to provide correct results, sensors must be calibrated to offer accurate readings. Despite limitations, sensor-based smart farming has the potential to revolutionize traditional farming by making it more precise and ecofriendly.

## 7. Conclusions

Smart farming and precision agriculture are important to boost food security throughout the world. Over the years, agriculture has undergone a series of transformations from field to customized growth chambers which provide a precisely calibrated and controlled environment, which is essential for healthy plant growth as well as for testing and adjusting the environmental parameters [135,136] There exist numerous biophysical instruments that can automatically track, monitor, and analyze a plant’s growth and its environmental conditions [137]. With the advent of sensors, real- time monitoring of environmental stressors and plants’ responses has transformed traditional farming into more precise and smart farming. Sensors (biosensors and nanosensors) play a multifaceted role in plant research such as detection of signaling molecules and environmental stressors. Smart agriculture is rapidly transforming traditional agricultural production practices across the globe. This transformation, based on technical innovation, is critical to ensuring a viable food production. One of the primary components of smart agriculture is sensors. The use of nanosensors or biosensors in plant science provides valuable insights into the role of different signaling molecules at different cellular compartments which allows researchers to explore their in vivo distribution and transport as well as reactions to environmental variables. Sensors enable the rewiring and understanding of dynamic networks of complicated plant signal transduction and metabolism at any organizational size which play a key role in targeted metabolic engineering for crop improvement. Under field conditions, sensors are used for nutrient analysis to determine whether fortification is necessary for optimal plant development, as well as to detect infections. Sensors may give a wide range of information, allowing farmers to better care for their crops and fields. Farmers can construct a holistic image of their farms, crops, and fields by gathering a variety of data via sensors, allowing them to prepare for the future. They know which fields require special attention, which are ready for crops, which crops did well with the weather, and so on. From there, they are empowered to make plans for the future. Sensors can be used to determine soil pH, soil moisture levels, soil compaction, soil composition, weed identification, the condition of farming equipment, and even weather. Owing to their multifaceted roles, sensors can reform traditional agriculture into precise smart farming which can help to track crop performance in real time under changing environmental conditions.

## Figures and Tables

**Table 1:** The applications of nanosensors for pesticide detection.

Nanosensor Type	Sensor Types and Sensing Mechanism	Pesticide Detected and Trace Amounts	Purpose	Finding	References
Fluorescent-nanosensor	Imazapyr quenches the fluorescence intensity of aminopropyltriethoxysilane (APTES)-coated ytterbium oxide (Yb_2_O_3_) nanoparticles.	Imazapyr at 0.2 ppm	The hydrothermal production of ytterbium oxide (Yb_2_O_3_) nanoparticles was followed by surface modification with aminopropyltriethoxysilane (APTES) to create a biocompatible tunable fluorescent nanosensor for the accurate and effective monitoring of imazapyr.	Exhibited excellent efficiency in detecting imazapyr and demonstrating its potential for herbicide sensing in real field conditions.	[56]
	Introduction of glyphosate into the solution leads to the inhibition of the catalytic activity of Copper (II) oxide (CuO) by multiwall carbon nanotube (MWCNT) nanomaterials, resulting in a fluorescence response being turned off.	Glyphosate at 0.67 ppb	Turn-off fluorescence sensor that detects glyphosate by inhibiting the catalytic activity of CuO/MWCNTs.	A highly efficient and sensitive nanosensor for detecting glyphosate.	[62]
2. Surface plasmon resonance (SPR)	Affinity sensor. Atrazine selectively binds to molecular imprinted nanoparticles on the gold surface of the SPR chip.	Atrazine at 0.7134 ng/mL	Atrazine-imprinted nanoparticles are synthesised using the emulsion polymerization process and subsequently affixed to the gold surface of the surface plasmon resonance system.	Selective atrazine detection using plastic antibody-based surface plasmon resonance nanosensors.	[68]
	Optic-sensor: interaction with silver film leading to change in refractive index.	Fenitrothion at 38 nM	Fenitrothion is determined by utilizing Ta_2_O_5_ nanostructures immobilized onto a reduced graphene oxide matrix.	Use of selective and sensitive optical fiber sensor utilizing SPR for the identification of fenitrothion pesticide.	[57]
3. Electrochemical sensor	Luminescence sensor: glyphosate inhibits enzymatic reaction by competing with L-cysteine which in turn forms glyphosate-Cu (II). This complex inhibits the catalytic action of peroxidase-mimicking substances.	Glyphosate at 0.5 nM	An electrochemical luminescence sensor employing a double suppression mechanism for the highly sensitive detection of glyphosate.	The sensor detects glyphosate using a double inhibition approach with effective detection performance, accurate sensitivity, reproducibility and stability in detection of glyphosate.	[63]
	Electrochemical detection of methyl parathion using CuO-TiO_2_ complex nanocomposites coupled with a glass carbon electrode.	Methyl parathion at 1.21 ppb	Efficient detection of methyl parathion pesticide using non-enzymatic electrochemical sensor based on CuO-TiO_2._	Using non-enzymatic electrochemical nanosensor with CuO-TiO_2_ hybrid nanocomposites for sensitive and selective detection of methyl parathion.	[64]
	Aptasensor based sensor. Specific interaction between the biotinylated aptamer sequence of DNA and malathion molecules, immobilized onto the iron oxide-doped chitosan/FTO electrode.	Malathion at 0.001 ng/mL	Efficient sensors for the detection of malathion which provide a rapid and reliable method for analyzing malathion contamination in lettuce leaves and soil samples.	The successful fabrication and characterization of chitosan–iron oxide nanocomposite (CHIT–IO) layer on fluorine tin oxide (FTO) electrode as well as the detection of malathion in lettuce leaves and soil sample.	[65]
	This nanosensor relies on the hindrance of the redox reaction of CuO nanoparticles by malathion.	Malathion at 0.01 nM	To provide an efficient electrochemical platform for the identification of malathion, utilizing copper oxide nanoparticles supported on 3D graphene as a non-enzymatic sensing interface.	In soil sample, malation detection was based on copper oxide nanoparticles supported by three-dimensional graphene used by the electrochemical sensor.	[69]
4. Optical nanosensor	The strength of the surface-enhanced Raman spectroscopy (SERS) signal rises accordingly with the concentration of dimethoate.	Dimethoate at 0.002 ppm	Surface-enhanced Raman spectroscopy (SERS) using silver nanodendrites on microsphere end-shape optical fibre for the identification of pesticide residues.	Enabling highly sensitive identification of Rhodamine-6-G and dimethoate pesticide at ultralow concentrations, demonstrating its potential for highly-sensitive chemo-sensing applications.	[66]
	To detect variations in the concentration of metribuzin, the distinctive luminescent capabilities of upconverting nanoparticles (UCNPs) are combined with the colorimetric response of a near infrared (NIR) dye contained in a polyvinyl chloride (PVC) matrix.	Metribuzin at 6.8 × 10^−8^ M	To enable the detection of metribuzin, a prevalent pesticide, within a low concentration range using a ratiometric and colorimetric optical sensor film.	Highly sensitive sensor with UCNPs’ distinctive luminous features and outstanding recognition abilities at extremely low detection limits.	[67]

**Table 2:** The roles of nanosensors in the detection of heavy metals.

Nanosensor Type	Sensor Types and Sensing Mechanism	Detected Heavy Metal and Trace Amounts	Purpose	Finding	References
ICTS nanosensor	Monoclonal antibodies bind specifically to the cadmium-ethylenediaminetetraacetic acid (EDTA) complex, allowing for more selective detection of cadmium ions in aqueous samples.	Cadmium (Cd) at 0.35 µg/L	Using specific on-site screening tool utilizing an enhanced test strip for the quick identification of cadmium [Cd (II)] ions, particularly when the sample comprises the excess of ethylenediaminetetraacetic acid (EDTA)	Sensitive and specific colorimetric test strip that uses a monoclonal antibody for the cadmium-ethylenediaminetetraacetic acid (EDTA) complex, capable of detecting cadmium.	[95]
Colorimetric nanosensor	Mn_3_O_4_ nanoparticles’ oxidase-mimicking activity via oligonucleotides, where heavy metal ions interfere with the inhibition of tetramethylbenzidine (TMB) oxidation, resulting in a color change from light green to yellow, allowing visual identification of heavy metal ions in solution.	Mercury (Hg (II)) at 3.8 μg·L−1 and cadmium [Cd (II)] at 2.4 μg·L−1	A colorimetric test that uses Mn_3_O_4_ nanoparticles regulated by oligonucleotides to visually identify heavy metals, specifically mercury [Hg (II)] as well as cadmium [Cd (II)], with the aim of obtaining high sensitivity and selectivity.	Colorimetric technique using Mn_3_O_4_ nanoparticles regulated by oligonucleotides for visual detection of heavy metals, particularly mercury [Hg (II)] as well as Cd (II), with good sensitivity, selectivity, and validity in water samples.	[96]
	Etching silver-coated gold nanobipyramids causes a color shift that is used to detect Hg^2+^.	Mercury at 0.8 µM	The gold nanobundles Au NBs were created using the seed-mediated growth method, and then different quantities of AgNO_3_ were added to the colloidal solution to form Au NBs–Ag nanoparticles. The Au NBs were created using the seed-mediated growth method, and then different quantities of AgNO_3_ were added to the colloidal solution to form Au-NBs–Ag nanoparticles.	The strategy saves time and eliminates the need for difficult operations.	[97]
	Hg (II) ions coupled with the dithioacetal-based stimulus–responsive molecular gates cause a colorimetric shift in the reporter dye placed onto the mechanized mesoporous silica nanoparticles (MSN), allowing for sensitive and selective detection of Hg (II) ions.	Mercury (Hg) at 60 pM	A highly efficient colorimetric nanosensor for detecting Hg (II) ions, using mechanized mesoporous silica nanoparticles functionalized with stimulus-responsive molecular gates.	Hg (II) is detected using a colorimetric nanosensor that uses mechanized mesoporous silica nanoparticles functionalized with dithioacetal-based molecular gates.	[98]
	Pd (II) aggregated APP-AuNPs more readily than other metals, thereby eliminating the SPR.	Palladium Pd (II) at 4.23 µM	To detect Pd(II), gold nanoparticles were stabilized using the cationic 1-(3-(acetylthio)propyl)pyrazin-1-ium ligand.	The nanosensors permit naked eye detection.	[96]
Optical nanosensor	Nanohybrid CdSe QDs. Following the addition of cadmium, green photoluminescence gradually returned.	Cadmium at 25 nM	Utilizing a modified reverse microemulsion technique, amino-capped CdTe–SiO_2_ core-shell-structured fluorescent silica nanoparticles were created. The CdTe–SiO_2_–CdSe ratiometric probes were made by covalently pairing green-emitting dual-stabilizer-capped CdSe to the silica membrane.		[96]
Multimodal nanosensor	Fluorescence quenching as the quantity of Hg^2+^ increases.	Mercury at 0.49 nM	Following their preparation using the chemical coprecipitation process, silica-coated Fe_2_O_3_ nanoparticles were electrostatically bonded to cysteamine-capped CdTe QDs.	The identified analyte can be eliminated with an external bar magnet, leaving no residual contamination.	[99]
Surface plasmon resonance	When the metal bound to silver nanoparticles based on epicatechin, it displayed a hyperchromic change.	Lead at 1.52 μM	The epicatechin and AgNO_3_ ratios were mixed, and then the mixture was stirred magnetically to create the ECAgNPs, which were then employed for lead detection.	AgNPs can preferentially detect Pb^2+^ in the presence of additional interfering metal ions.	[100]
Electrochemical sensor	As heavy metal concentrations rise, the peak current rises as well.	Cadmium at 8.5 nM, lead at 0.6 nM and copper at 0.8 nM	N-hydroxysuccinimide (NHS) and 1-ethyl-3-(3-dimethylaminopropyl) carbodiimide (EDC) were used as crosslinking agents to prepare Fc-NH_2_-UiO-66, which was then dispersed on the trGNO nanosheets, and NH_2_-UiO-66 which was synthesized hydrothermally.	Found to be an excellent platform for the identification of numerous heavy metal ions at once.	[96]
Magnetic-fluorescent based nanosensor	Quenching of nanosensor’s fluorescence.	Mercury at 9.1 × 10^−8^ mol/L	Fe_3_O_4_ nanoparticles and QDs were encapsulated using carboxymethyl chitosan as an encapsulating agent, producing multifunctional magnetic–fluorescent nanoparticles that were subsequently employed as nanosensors.	The nanosensor exhibits improved Hg^2+^ ion selectivity and sensitivity.	[101]
